# Eleutheroside B Protects against Acute Kidney Injury by Activating IGF Pathway

**DOI:** 10.3390/molecules24213876

**Published:** 2019-10-28

**Authors:** Hongmei Zang, Qin Yang, Jun Li

**Affiliations:** 1 School of Pharmacy, Anhui Medical University, Hefei 230032, China; zanghongmei@ahmu.edu.cn (H.Z.); yangqin940812@163.com (Q.Y.); 2 Anhui Institute of Innovative Drugs, Hefei 230032, China; 3 Key Laboratory of Anti-Inflammatory and Immune Medicine, Ministry of Education, Hefei 230032, China

**Keywords:** eleutheroside B, acute kidney injury, cisplatin, IGFBP-7, programmed necroptosis, inflammation, apoptosis

## Abstract

Acute kidney injury (AKI) is a common, complex, and severe clinical syndrome characterized by rapid decline in renal function, combined with tissue damage. Currently, the prevention and treatment of AKI are focused on symptomatic treatment, rather than treating the underlying causes. Therefore, there is no specific treatment to prevent renal injury except for renal dialysis. In this study, we used cisplatin-induced AKI mouse and human kidney-2 (HK-2) cell models to evaluate the renal protective effect of eleutheroside B, an active compound in traditional Chinese medicines. MTT assay was used to detect the effect of eleutheroside B on proliferation of human HK-2 cells in presence and in absence of cisplatin. Western blot and immunostaining were used to detect the protein level of kidney injury molecule-1 (KIM-1), cleaved caspase-3, receptor-interacting protein kinase (RIPK)-1, and RIPK-3. Real-time PCR was used to detect the mRNA levels of chemokines (like monocyte chemotactic protein 1, MCP-1) and pro-inflammatory cytokines including interleukin-6 (IL-6) and tumor necrosis factor (TNF-α). Flow cytometry assay was used to detect apoptosis of HK-2 cells. In vivo results showed that eleutheroside B reduced the increase in serum creatinine and blood urea nitrogen (BUN) levels in the AKI model. Periodic acid-Schiff staining and Western blot analysis of KIM-1 showed that eleutheroside B alleviated tubular cell injury. Further, eleutheroside B reduced macrophage infiltration and production of inflammatory cytokines, inhibited the activation of nuclear factor (NF)-κB, and inhibited apoptosis and programmed necrosis. The mechanism may be that eleutheroside B can activate the insulin-like growth factor (IGF) pathway and its downstream pathway by downregulating the expression of IGFBP-7, thus promoting cell proliferation. Therefore, our results suggest that eleutheroside B is a potential drug for AKI treatment.

## 1. Introduction

Acute kidney injury (AKI) is a common, complex, and severe clinical syndrome characterized by the rapid decline of renal function combined with tissue injury [1,2,3,4]. AKI generally occurs after acute or chronic illness. According to the world epidemiological survey, the incidence of AKI in hospitalized patients is 21%, and the mortality rate of AKI patients requiring dialysis treatment is 50% [5]. AKI occurs in patients with or without underlying chronic kidney disease (CKD). Incomplete recovery may lead to new or CKD [4,6]. Percutaneous coronary intervention, cardiac surgery, liver surgery, and vascular surgery are associated with higher AKI risk [7,8,9].

Currently, the prevention and treatment of AKI are mainly symptomatic, focusing on timely correction of reversible causes, intervention treatment, maintaining the stability of the physiological environment of the kidney, effective control of infection, nutritional support, and the prevention and treatment of complications. No drugs for the prevention and treatment of AKI have been recommended by Kidney Disease Improving Global Outcomes (KDIGO) [10]. It is urgent to translate the findings of basic research into clinical practice to improve the outcome of patients. Therefore, in recent years, researchers have focused on discovering new drugs for the prevention of AKI. Currently, the drugs used for the treatment and prevention of AKI are divided into inducing an anti-inflammatory response [11,12], the inhibition of apoptosis [13,14], and the promotion of cell proliferation [15].

Eleutheroside B is a phenolic glycoside which exists in various medicinal plants, including *Acanthopanax senticosus* [16]. Eleutheroside B has been observed to have a variety of pharmacological activities, including anti-inflammatory and anti-radiation [17,18,19,20]. In traditional Chinese medicine, *A. senticosus* is used to treat deficiency of the kidney. Eleutheroside B is the major bioactive component of this herb. We believe that eleutheroside B can be used to prevent and treat kidney diseases. In this study, we evaluated the protective effect of eleutheroside B on ischemia-reperfusion induced AKI and cisplatin-induced AKI in mice and human kidney-2 (HK-2) cells and discussed its possible mechanism.

## 2. Results

### 2.1. Effect of Eleutheroside B on Cisplatin-Induced Toxicity and Injury in HK-2 Cells

**Figure 1:**
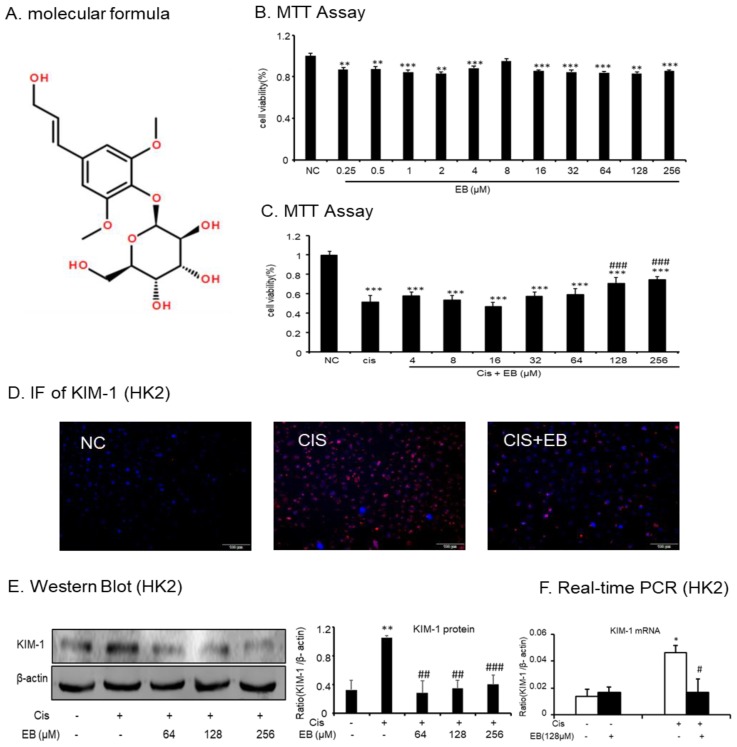
Effect of eleutheroside B on cell viability and kidney injury molecule-1 (KIM-1) levels with or without cisplatin treatment. (**A**) Molecular structure of eleutheroside B. (**B**) Effect of different concentrations of eleutheroside B on viability of human kidney-2 (HK-2) cells by MTT assay. (**C**) Eleutheroside B restored cell viability in cisplatin-treated HK-2 cells by MTT assay. (**D**) Immunofluorescence of KIM-1 in HK-2 cells. Eleutheroside B treatment significantly reduced the protein level of KIM-1 in cisplatin-treated HK-2 cells. (**E**) Western blot analysis of KIM-1 protein in HK-2 cells. (**F**) Real-time PCR in HK-2 cells. Eleutheroside B significantly decreased cisplatin-induced mRNA level of KIM-1. Data represent the mean ± SEM for 3–4 independent experiments in vitro. ** *p* < 0.05, ** *p* < 0.01, *** *p* < 0.001 versus normal; ^#^ *p* < 0.05, ^##^ *p* < 0.01, ^###^ *p* < 0.001 compared to cisplatin-treated group. Cis, cisplatin.

The effects of different concentrations of eleutheroside B (Figure 1A) (0.25, 0.5, 1, 2, 4, 8, 16, 32, 64, 128, and 256 μM) on HK-2 cells were examined by MTT. Within the concentration range of 0.25–256 μM, the effect of eleutheroside B on cell viability was not obvious, indicating that the toxicity of eleutheroside B was low (Figure 1B). In addition, eleutheroside B at concentrations of 128 and 256 μM significantly (^###^ *p* < 0.001) reduced the effect of cisplatin (20 μM) on cell viability (Figure 1C).

The immunofluorescence (IF) assay was used to detect the expression of kidney injury molecule-1 (KIM-1) in each group. The red fluorescence of KIM-1 in normal cells was low. The expression of KIM-1 in the cisplatin-stimulated model group was significantly increased but this was significantly decreased by eleutheroside B (Figure 1D). Western blot and real-time PCR were used to investigate the expression of KIM-1 protein and mRNA in the HK-2 cells of each group. Cisplatin upregulated the expression of KIM-1 protein (** *p* < 0.01) and mRNA (* *p* < 0.05) in HK-2 cells. However, different concentrations of eleutheroside B at 64, 128, and 256 μM significantly reduced the protein expression of KIM-1 (^##^ *p* < 0.01, ^##^ *p* < 0.01, ^###^ *p* < 0.001, respectively) (Figure 1E,F).

### 2.2. Effects of Eleutheroside B on Inflammatory Cytokines Induced by Cisplatin in HK-2 Cells

**Figure 2:**
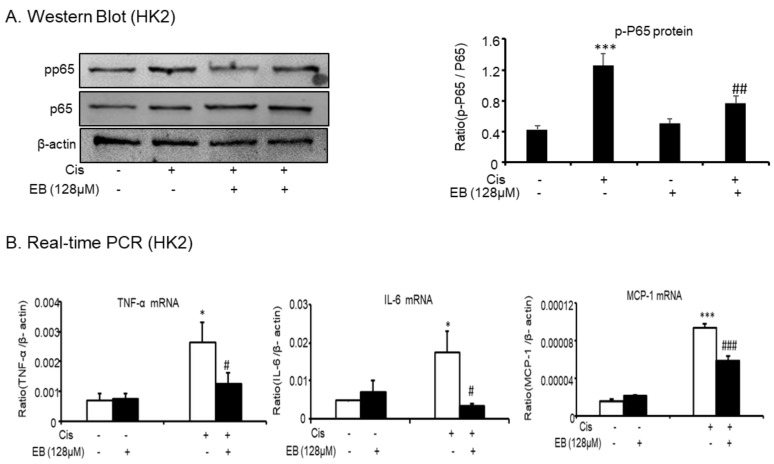
Eleutheroside B reduced cisplatin-induced inflammatory response in HK-2 cells. (**A**) Western blot analysis p-P65 in cisplatin-treated HK-2 cells. (**B**) Real-time PCR in HK-2 cells. Results demonstrate that eleutheroside B largely reduced cisplatin-induced mRNA levels of tumor necrosis factor (TNF)-α, monocyte chemotactic protein 1, and interleukin-6 (IL-6). Data represent the mean ± SEM for 3–4 independent experiments. * *p* < 0.05, *** *p* < 0.001 compared with control; ^#^ *p* < 0.05, ^##^ *p* < 0.01, ^###^ *p* < 0.001 compared with cisplatin-treated group. Cis, cisplatin; EB, eleutheroside B.

Cisplatin-induced inflammation caused an increase in the levels of a variety of inflammatory cytokines via the activation of the nuclear factor κB (NF-κB) intracellular signaling pathways. The activation of NF-κB in the classical inflammatory pathway is associated with the inflammatory response of AKI, thus the detection of NF-κB is necessary. Western blot analysis showed that eleutheroside B reduced the expression of cisplatin-induced activated p-P65 (^##^ *p* < 0.01) (Figure 2A). To further study the inhibitory effect of eleutheroside B on the cisplatin-induced inflammatory response, real-time PCR was used to detect multiple inflammatory factors (TNF-α, IL-6) and monocyte chemotactic protein 1 (Figure 2B) in HK-2 cells. Eleutheroside B significantly downregulated the expression of cisplatin-induced inflammatory factors (TNF-α, IL-6) (^#^ *p* < 0.05) and chemokines (^###^ *p* < 0.001). These results suggest that eleutheroside B (128 μΜ) reduced cisplatin-induced inflammatory cell infiltration, reduced the expression of inflammatory factors and chemokines, and inhibited the inflammatory response in vitro.

### 2.3. Effects of Eleutheroside B on Apoptosis and Necroptosis Caused by Cisplatin in HK-2 Cells

**Figure 3:**
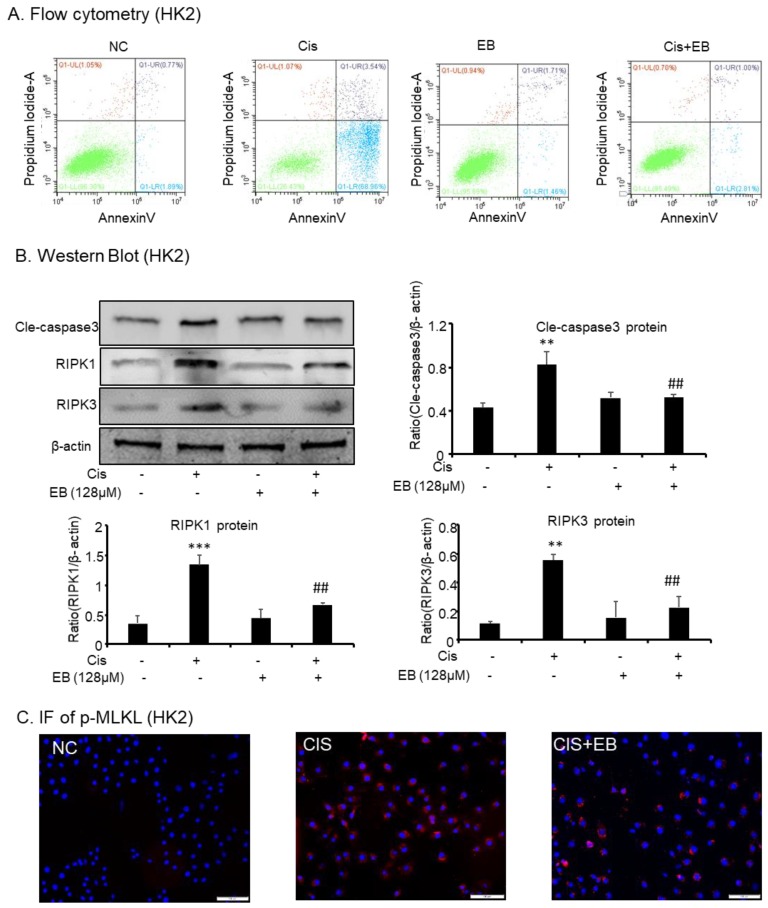
Eleutheroside B attenuated programmed cell death induced by cisplatin in HK-2 cells. (**A**) Flow cytometry in HK-2 cells. (**B**) Western blotting analysis of programmed cell death-related molecules. (**C**) Immunofluorescence of phosphorylated-mixed lineage kinase domain like in HK-2 cells. Eleutheroside B significantly downregulated receptor-interacting protein kinase (RIPK)1, RIPK3, p-mixed lineage kinase domain-like protein (p-MLKL), and apoptosis-related protein cle-caspase3 in cisplatin-treated HK-2 cells. Data represent the mean ± SEM for 3–4 independent experiments in vitro. ** *p* < 0.01, *** *p* < 0.001 compared to normal; ^##^ *p* < 0.01 compared to model. Cis, cisplatin; EB, Eleutheroside B.

Fluorescein isothiocyanate (FITC)-labeled Annexin V was used as a probe to label phosphatidylserine (PS), which is flipped out of the cell membrane during the apoptotic process, and phosphatidylinositol (PI) labeled the nucleus. Flow cytometry was used to detect the apoptotic status of each group. The proportion of early apoptotic and late apoptotic/necrotic HK-2 cells stimulated by cisplatin increased from 1.89% and 0.77% to 68.96% and 3.54%, respectively. Compared with the model group, eleutheroside B significantly reduced the apoptosis and necrosis of HK-2 cells induced by cisplatin (Figure 3A). Western blot and IF were used to detect the expression of apoptotic and necroptosis related proteins in HK-2 cells. The expression of receptor-interacting protein kinase (RIPK)1 (*** *p* < 0.001), RIPK3 (** *p* < 0.01), downstream p-mixed lineage kinase domain-like protein (p-MLKL), and apoptotic-related protein Cle-caspase-3 (** *p* < 0.01) were upregulated by cisplatin stimulation but decreased after eleutheroside B treatment (^##^ *p* < 0.01) (Figure 3B,C).

### 2.4. Effect of Eleutheroside B on Cisplatin-Induced Renal Dysfunction and Histopathology

**Figure 4:**
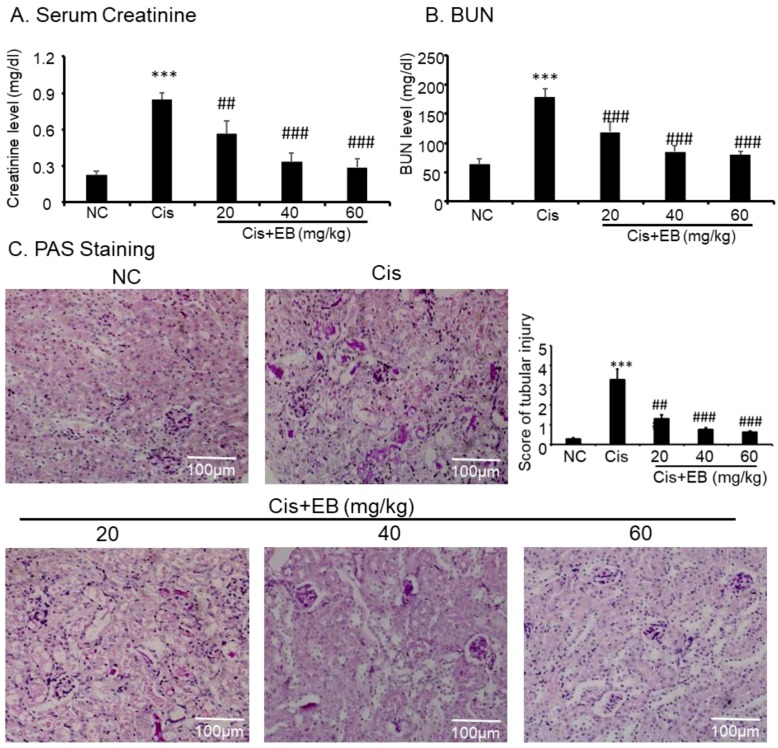
Changes in the levels of (**A**) serum creatinine, (**B**) blood urea nitrogen (BUN), and (**C**) histopathology after an intraperitoneal injection of cisplatin (20 mg/kg). Saline or eleutheroside B was injected 30 min before the injection of cisplatin. Each column represents the mean ± SD (n = 4–7). Results of serum creatinine and BUN show that treatment of eleutheroside B (20, 40, 60 mg/kg) restored renal function in cisplatin nephropathy. C: periodic acid-Schiff (PAS) staining and score. Results of PAS staining and score of severity indicate that treatment with set concentrations of eleutheroside B (20, 40, 60 mg/kg) alleviated tubular necrosis, tubular dilation, and cast formation in cisplatin nephropathy. Data are mean ± SEM for 6–8 mice. *** *p* < 0.001 compared to normal; ^##^ *p* < 0.01, ^###^ *p* < 0.001 compared to model. Cis, cisplatin; EB, eleutheroside B. KIM-1: kidney injury molecule-1. Magnification, 20×.

In order to investigate the effect of eleutheroside B on renal function, creatinine and blood urea nitrogen (BUN) levels were detected by a detection kit. Creatinine and BUN levels in the serum of mice in the model group were significantly higher than those in control group (*** *p* < 0.001). However, creatinine and BUN levels were significantly lower (^###^ *p* < 0.001) in the serum of model mice with different doses of eleutheroside B (Figure 4A,B). Cisplatin was used to induce AKI in C57BL/6 mice, and eleutheroside B was given to investigate the effects on renal histopathology. The results of periodic acid-Schiff (PAS) staining (×20) showed that the structure of renal tubules in the control group was normal and glycogen deposition was not seen in the field of vision. PAS also revealed that the thickness of renal tubules in the model group became thinner, the shape of the tubular lumen changed, cells were arranged in a disorderly fashion or were partially absent, and glycogen deposition increased. Eleutheroside B significantly reduced renal tubule damage and glycogen deposition. Additionally, the deformation and arrangement of renal tubular cells were turbulent. After eleutheroside B treatment, the damage score of kidney tissues in middle-dose group (40 mg/kg) and high-dose group (60 mg/kg) decreased significantly (^###^ *p* < 0.001) (Figure 4C).

### 2.5. Effect of Eleutheroside B on Inflammation, Programmed Necrosis, and Apoptosis in Mice with AKI Induced by Cisplatin

**Figure 5:**
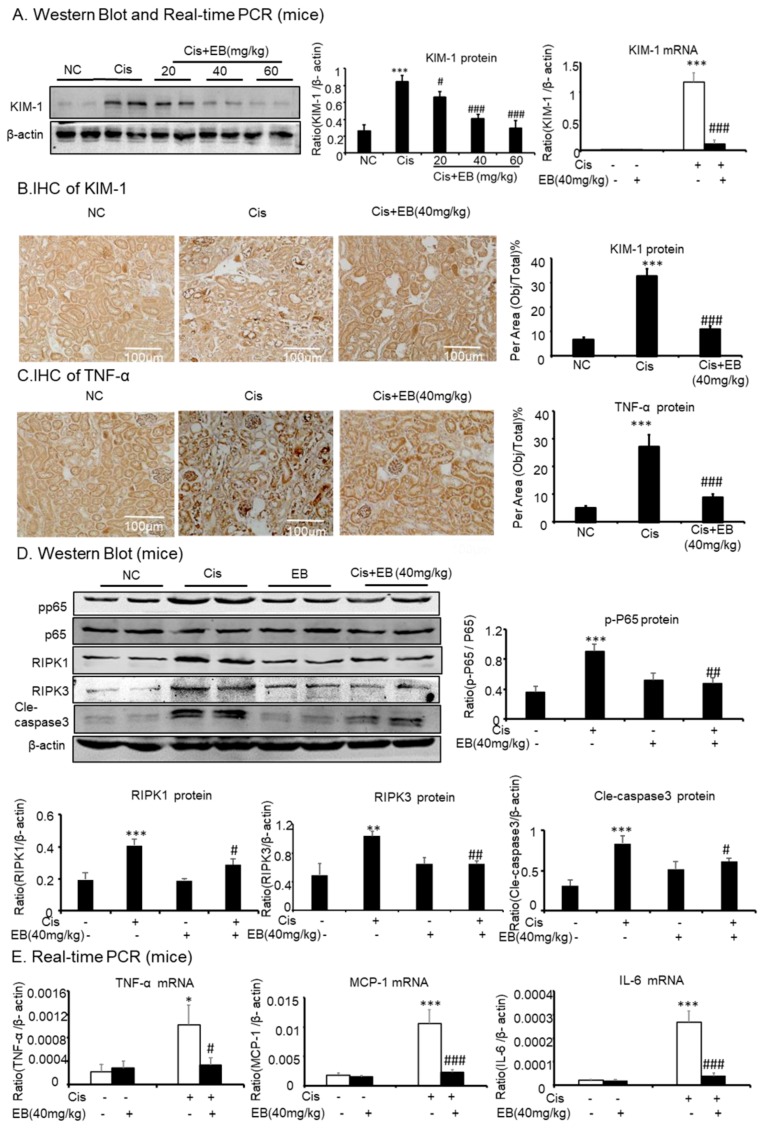
Eleutheroside B attenuated cisplatin-induced renal injury, inflammation, programmed necrosis, and apoptosis in vivo. (**A**) Western blot and real-time PCR analysis of KIM-1. (**B**) and (**C**) Immunohistochemistry of KIM-1 and TNF-α. IHC result and quantitative data indicate that treatment of eleutheroside B reduced the percentage of KIM-1 and TNF-α in injured kidney. (**D**) Western blot analysis and quantitative data of RIPK1, RIPK3, p-P65, and cle-caspase-3. (**E**) Real-time PCR of inflammation indexes. Real-time PCR demonstrate that treatment of eleutheroside B largely blocked upregulated mRNA levels of MCP-1, TNF-α, and IL-6 in cisplatin-injured kidney. * *p* < 0.05, ** *p* < 0.01, ** *p* < 0.001 compared to normal; ^#^ *p* < 0.05, ^##^ *p* < 0.01, ^###^ *p* < 0.001 compared to model. Cis, cisplatin; EB, eleutheroside B (40 mg/kg). KIM-1: kidney injury molecule-1. Magnification, 20×.

Western blot, real-time PCR, and immunohistochemistry (IHC) were used to detect the expression of KIM-1 in mice. Cisplatin significantly increased the expression of KIM-1 protein (*** *p* < 0.001) and mRNA (*** *p* < 0.001) levels in the model group compared with the control group. The middle (40 mg/kg) and high doses (60 mg/kg) of eleutheroside B significantly reduced the expression of KIM-1 (^###^ *p* < 0.001) in mice (Figure 5A,B). IHC showed that cisplatin significantly upregulated the expression of TNF-α (*** *p* < 0.001) in the model group, compared with the control group, and eleutheroside B significantly downregulated the expression of TNF-α at protein level (^###^ *p* < 0.001) (Figure 5C). Real-time PCR showed that eleutheroside B downregulated the expression of TNF-α (^#^ *p* < 0.05), IL-6 (^###^ *p* < 0.001), and MCP-1 (^###^ *p* < 0.001) in the model group. In addition, eleutheroside B reduced the level of p-P65 (^##^ *p* < 0.01), thereby inhibiting the NF-κB signaling pathway in vivo (Figure 5D, E). Western blot showed that eleutheroside B reduced protein levels of RIPK1 (^#^ *p* < 0.05) and RIPK3 (^##^ *p* < 0.01), and reduced the level of cisplatin-induced cle-caspase-3 (^#^ *p* < 0.05) in AKI, suggesting that eleutheroside B inhibited apoptosis and programmed necrosis in vivo (Figure 5E).

### 2.6. Effect of Eleutheroside B on KIM-1, Inflammation, Necrosis, and Apoptosis in Hypoxia Reoxygenation Injury-Induced (HRI-Induced) HK-2 Cells

**Figure 6:**
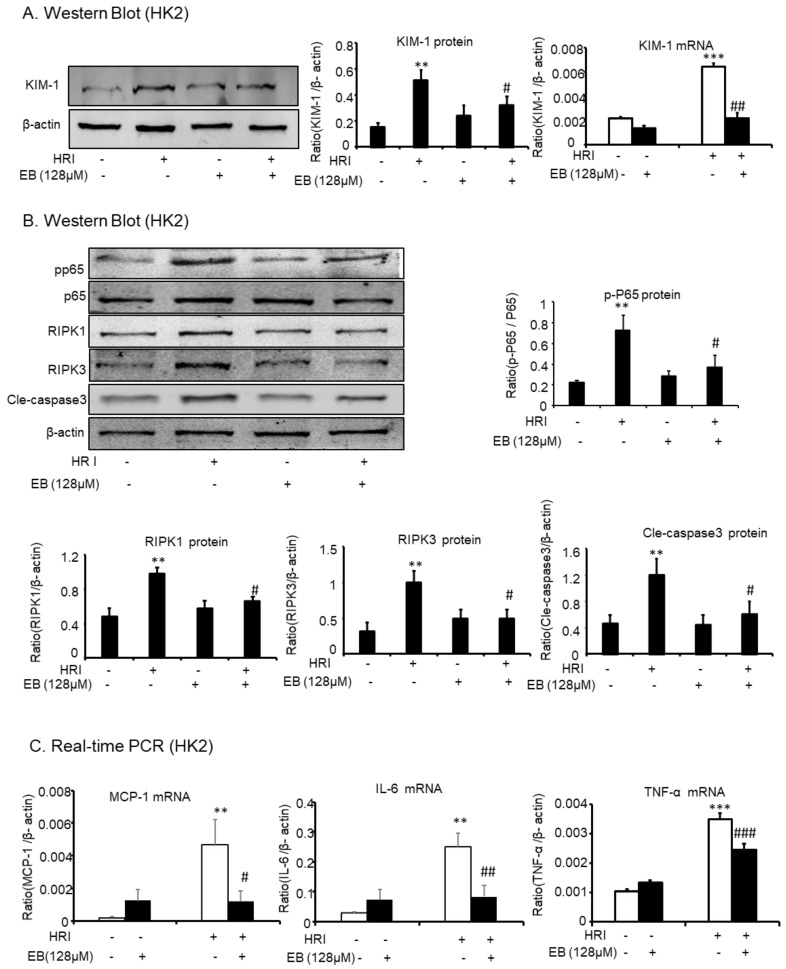
Eleutheroside B attenuated inflammation, programmed necrosis, and apoptosis in HR-induced HK-2 cells. (**A**) Western blot and real-time PCR analysis of KIM-1. (**B**) Western blot analysis of RIPK1, RIPK3, p-P65, and cle-caspase-3 in HR-induced HK-2 cells. Western blot result and quantitative data indicate that treatment of eleutheroside B reduced the percentage of KIM-1, RIPK1, RIPK3, p-P65, and cle-caspase-3 cells in HR-induced HK-2 cells. (**C**) Real-time PCR in HK-2 cells. Results demonstrate that eleutheroside B largely reduced HR-induced mRNA levels of TNF-α, MCP-1, and IL-6. Data represent the mean ± SEM for 3–4 independent experiments. ** *p* < 0.01, *** *p* < 0.001 compared with control; ^#^ *p* < 0.05, ^##^ *p* < 0.01, ^###^ *p* < 0.001 compared with cisplatin-treated group. HRI, hypoxia-reoxygenation injury; EB, eleutheroside B.

HRI-induced AKI experiments were used to further demonstrate the protective effect of eleutheroside B on AKI. Western blot showed that eleutheroside B downregulated KIM-1 (^#^ *p* < 0.05) induced by anoxic conditions in HK-2 cells (Figure 6A). In addition, eleutheroside B reduced the protein levels of p-P65 (^#^ *p* < 0.05), RIPK1 (^#^ *p* < 0.05), RIPK3 (^#^ *p* < 0.05), and cle-caspase-3 (^#^ *p* < 0.05) in HK-2 cells under anoxic conditions (Figure 6B). Furthermore, real-time PCR showed that eleutheroside B alleviated inflammation induced by anoxic conditions via the decrease of KIM-1 (^##^ *p* < 0.01), MCP-1 (^#^ *p* < 0.05), IL-6 (^##^ *p* < 0.01), and TNF-α (^###^ *p* < 0.001) (Figure 6C).

### 2.7. RNA-Seq Analyses

**Figure 7:**
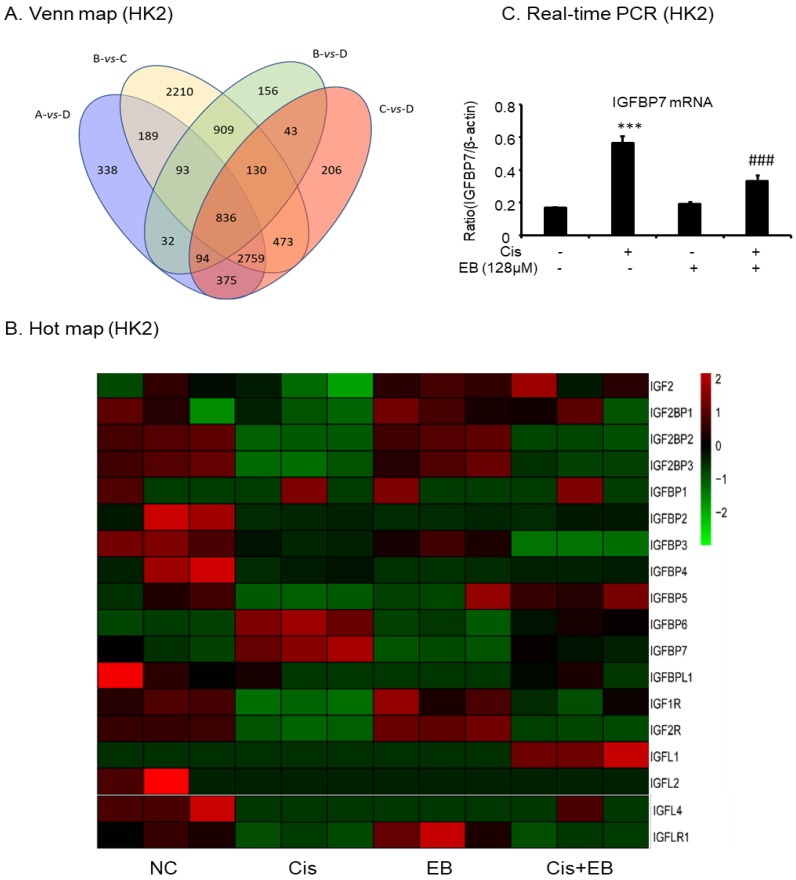
RNA-seq analysis results and verification. (**A**) Venn map of the number of significantly differentially expressed genes. (**B**) Hot map of gene expression related to insulin-like growth factor pathway. (**C**) Real-time PCR of IGFBP-7. Real-time PCR demonstrate that treatment of eleutheroside B largely blocked upregulated mRNA levels of IGFBP-7. *** *p* < 0.001, ^###^ *p* < 0.001.

RNA-Seq was used to screen the differentially expressed genes after eleutheroside B treatment. There were 7249 differentially expressed genes between groups A and B, 71 differentially expressed genes between groups A and C, 4716 differentially expressed genes between groups A and D, 7599 differentially expressed genes between groups B and C, 2293 differentially expressed genes between groups B and D, and 4916 differentially expressed genes between groups C and D (Figure 7A). Eleutheroside B activated the insulin-like growth factor (IGF) pathway by downregulating the expression of IGFBP-3 and IGFBP-7, and upregulating the expression of insulin-like growth factor receptor 1 (IGF1R), thereby activating the downstream pathway, promoting cell proliferation and exerting renal protection (Figure 7B). Real-time PCR showed that eleutheroside B downregulated the expression of IGFBP-7 in cisplatin-stimulated HK-2 cells (^###^ *p* < 0.001) (Figure 7C).

## 3. Discussion

The clinical symptoms of AKI are a sharp decline in function accompanied by tissue damage, and the main method of treatment is symptomatic treatment. Cisplatin is a platinum-based chemotherapeutic agent that often causes serious side effects, limiting its clinical application. After a single dose of 50–100 mg/m^2^, approximately 33% of patients develop renal toxicity [21,22]. Cisplatin-treated cell and animal models are the classical models for the study of AKI.

KIM-1 is a biomarker of AKI, which represents the degree of renal injury. The decrease of KIM-1 expression indicates the decrease of renal injury [23]. An inflammatory cascade reaction is one of the main causes of renal injury caused by cisplatin [24]. Experimental studies of AKI often use models such as ischemia and reperfusion injury-, drug nephrotoxicity, and sepsis endotoxemia [25], all of which involve inflammation [11,26,27]. Apoptosis, or programmed necrosis, of renal tubular epithelial cells is a common pathological feature in AKI models [13,14]. Apoptosis is a spontaneous, gene-controlled, and orderly cell death. It occurs spontaneously to maintain homeostasis after cells are triggered by external and internal environmental influences, or death signals [14]. Caspase activation plays an important role in apoptosis. Regulating the expression of apoptosis-related genes affects apoptosis in AKI models, thus affecting the occurrence and development of AKI [28,29]. Procedural necrosis, different from apoptosis and necrosis, is a programmed cell death that is regulated by genes but is not dependent on caspase pathways [30,31,32]. The key proteins for the initiation of this cell death are RIPK1 and RIPK3, which are composed of receptor-interacting protein kinase 1 (RIPK-1), receptor-interacting protein kinase 3 (RIPK-3), and MLKL. These components are phosphorylated by RIPK1 to form necrosis-inducing complexes, in which RIPK3 is recruited and phosphorylated by RIPK1. Phosphorylated MLKL triggers oligomerization and membrane translocation, leading to necrosis [28,29,33]. It has been reported that wogonin can inhibit the programmed necrosis of HK-2 cells induced by cisplatin in a RIPK1/RIPK3-dependent manner [12].

Currently, the prevention and treatment of AKI are mainly through anti-inflammatory, anti-oxidative stress, anti-apoptosis, and anti-programmed necrosis methods. In this study, we demonstrated that eleutheroside B reduced the cisplatin-induced increase of kidney injury molecule-1 (KIM-1), the expression of inflammatory factors (TNF-α, IL-6) and chemokines (MCP-1), the levels of apoptosis (cle-caspase-3), and programmed necrosis-related proteins (RIPK-1, RIPK-3, p-MLKL) in the human kidney-2 (HK-2) cell model. Eleutheroside B also reduced the levels of creatinine and urea nitrogen (BUN), inflammation, apoptosis, and programmed necrosis, and reduced the kidney damage caused by cisplatin in the AKI mouse model.

In addition to the above results, RNA-seq results showed that eleutheroside B could also reduce the expression of IGFBP-7 mRNA. As a molecular marker of acute kidney injury, IGFBP-7 has attracted much attention. However, little research has been done on its biological significance.

IGFs, including IGF1 and IGF2, bind with IGFRs to regulate somatic cell growth and proliferation in vivo [34]. The regulation process is regulated by IGFBPs. IGFBPs regulate the level of IGFs and have IGF-independent effects, including the inhibition of growth and promotion of apoptosis [35,36]. The activity of IGF1R is strictly regulated at various levels, such as production, endocytosis, and utilization of their homologous ligands [37,38]. The latter is partially controlled by IGFBPs. IGFBPs include six phenotypes with high affinity (IGFBP1–6) and nine phenotypes with low affinity (IGFBP-rP1–9) to IGFs. In addition, new IGFBP-related proteins [35,36], including IGFBP-7 also known as IGFBP-rP1, mac25, prostacyclin-stimulating factor (PSF), tumor adhesion factor (TAF), and angiopoietin (AGM), have been observed. Approximately 30% of the N-terminal domains (including conserved IGFBP motifs) from IGFBP1 to IGFBP6 are related to IGFBP-7. Therefore, IGFBP-7 is also considered to play a major role as a tumor suppressor [39]. Unlike IGFBP-1 to IGFBP-6, IGFBP-7 inhibits the proliferation of tumor cells by directly binding IGF1R [36,40].

There are two downstream pathways activated by the binding of IGF-1 and IGF-1R: the PI3K-Akt pathway [41] and the Ras/Raf/mitogen-activated extracellular signal-regulated kinase (MEK)/extracellular signal-regulated kinase (ERK) pathway [42,43]. The PI3K-Akt signaling pathway regulates the growth and differentiation of normal cells and the development of tumor cells. When exogenous substances such as IGF-1 and epidermal growth factor (EGF) stimulate cells, PI3K is activated, phosphatidylinositol 4,5-bisphosphate (PIP2) is phosphorylated into phosphatidylinositol (3,4,5)-trisphosphate (PIP3), and used as a second messenger to transduce signals to downstream Akt. The combination of Akt and PIP3 is activated to promote cell proliferation and anti-apoptosis [41]. Many extracellular factors, such as growth factors, adhesion molecules, differentiation factors, and tumor-promoting factors regulate cell behavior by Ras/Raf/MEK/ERK signaling pathway. Extracellular factors such as growth factors interact with their corresponding tyrosine kinases. GRB2 (growth factor receptor binding protein 2 adapter) recognizes phosphotyrosine docking sites on receptor or receptor substrate proteins and induces Ras activation through GDP/GTP exchange factor son of sevenless. Activated RAS binds to Raf serine/threonine kinases with high affinity and causes their translocation to cell membranes and activation. Activated Raf can activate serine/threonine kinase MEK1 and MEK2 through phosphorylation of two serine residues. Activated MEK activates serine/threonine kinase ERK1 and ERK2. Phosphorylated ERK can transfer to the nucleus, activate various transcription factors through phosphorylation, and regulate gene expression, thus promoting cell proliferation, differentiation, and survival [44,45].

It was reported that the expression of IGFBP-7 was increased in AKI and HK-2 cells induced by lipopolysaccharide. IGFBP-7 knockout can activate ERK1/2 signaling pathway, reduce creatinine, urea nitrogen, albumin, and cell apoptosis in mice urine, and effectively reduce the severity of renal injury, indicating that IGFBP-7 regulates AKI induced by sepsis through ERK1/2 signaling [46]. Here, we identified that the expression of IGFBP-7 increased in HK-2 cells with cisplatin induction, and eleutheroside B decreased the expression of IGFBP-7 in cisplatin-induced HK-2 cells.

In summary, the present study found that eleutheroside B protects against cisplatin-induced AKI in mice and alleviated the damage resulting from cisplatin exposure and hypoxia-reoxygenation in HK-2 cells. Eleutheroside B inhibits the expression of KIM-1, reduces inflammation, and prevents apoptosis and programmed necrosis. The mechanism may be that eleutheroside B can activate the IGF pathway and its downstream pathway by downregulating the expression of IGFBP-7, thus promoting cell proliferation. Therefore, our results suggest that eleutheroside B is a renal protective adjuvant for cisplatin-induced AKI.

## 4. Materials and Methods

### 4.1. Reagents and Materials

Eleutheroside B was purchased from Chengdu Herbpurify Biotechnology (2018041701, CAS 118-34-3, Chengdu, China). Cisplatin was purchased from Sigma-Aldrich (Sigma, St. Louis, MO, USA). Lactate dehydrogenase (LDH), 3-(4,5-dimethylthiazol-2-yl)-2,5-diphenyl-tetrazolium bromide (MTT), and Annexin V-FITC/PI Apoptosis Detection Kit was obtained from Beyotime (Shanghai, China). Periodic acid-Schiff (PAS), Creatinine Assay Kit, BUN Assay Kit, and Malondialdehyde (MDA) Assay Kit were purchased from Nanjing Jiancheng Bioengineering Institute (Nanjing, China). Dulbecco’s modified Eagle’s medium (DMEM), fetal bovine serum (FBS), and other cell culture reagents were obtained from Invitrogen. Antibodies specific to KIM-1, β-actin, RIPK1, and RIPK3 were purchased from Santa Cruz Biotechnology. Anti-P-MLKL and anti-cleaved-caspase3 were obtained from Cell Signaling Technology (CST, Danvers, MA, USA). IRDye 800-conjugated secondary antibody was purchased from Licor (Lincoln, NE, USA).

### 4.2. Murine Model of Cisplatin-Induced AKI

C57BL/6 mice (6–8 weeks old) were obtained from the Laboratory Animal Center of Anhui Medical University and placed in a specific pathogen-free facility. The experimental program and procedure were approved by the Animal Experimental Ethics Committee of Anhui Medical University. The model of AKI induced by cisplatin was established by intraperitoneal injection of cisplatin (single dose of 20 mg/kg) in mice, whereas the control group was injected with saline [12,27]. Eleutheroside B (20, 40, and 60 mg/kg) was injected intraperitoneally every day. The mice were anesthetized after 3 days, and kidney and blood were collected for further analysis. Blood was collected according to the manufacturer’s instructions to detect blood urea nitrogen (BUN) and creatinine. Kidney tissues were collected for paraffin embedding (Nanjing Jiancheng Institute of Bioengineering).

### 4.3. Cell Culture

Human tubular epithelial cells (HK-2) were cultured in a HyClone™ Dulbecco’s modified Eagle’s medium (DMEM)-F12 containing 5% fetal bovine serum (FBS) at 37 °C and 5% CO_2_. After starvation for 12 h with 0.5% FBS, the cells were pretreated with eleutheroside B and treated with cisplatin (20 μM) for 24 h. Hypoxia/reoxygenation (H/R) injury was used to mimic IRI *in vitro*. Briefly, cells were incubated in a glucose-free medium in a tri-gas incubator (94% N_2_, 5% CO_2_, and 1.0% O_2_) at 37 °C for 24 h. Subsequently, cells were incubated in a complete medium under normal conditions for 2 h for reoxygenation. Then, thiazole blue colorimetric method (MTT), real-time polymerase chain reaction (PCR), and western blot analysis were used to detect the cell survival rate, renal tubular injury index (indicated by kidney injury molecule-1 (KIM-1)), apoptosis (indicated by cle-caspase-3), necrosis index (indicated by receptor-interacting serine/threonine-protein kinase 1 (RIPK1), receptor-interacting serine/threonine-protein kinase 3 (RIPK3), and p-mixed lineage kinase domain-like protein (p-MLKL)), and expression of inflammatory factors (indicated by tumor necrosis factor-alpha (TNF-α), and interleukin 6 (IL-6)). Three to four independent experiments were conducted in vitro.

### 4.4. MTT Assay

MTT was used for detecting cell viability [12,27]. The enzymes in living cells reduce MTT to formazan and form a blue-violet precipitate. Logarithmically grown HK-2 cells were seeded in 96-well plates containing 200 μL of medium, and the number of cells per well was approximately 5 × 10^3^. After cell adherent growth, different concentrations of eleutheroside B were added and incubated for 12 h, with or without cisplatin (20 μM) for 24 h. After 24 h, 20 μL of 5 mg/mL MTT solution was added per well, protected from light, and incubated at 37 °C for 4 h. The supernatant was aspirated, 150 μL of dimethylsulfoxide (DMSO) was added to dissolve the formazan in the cells, and the optical density (OD) value was measured by an enzyme-linked immunosorbent detector at a wavelength of 492 nm.

### 4.5. Renal RNA Extraction and Real-Time PCR

According to the manufacturer’s instructions, total RNA was extracted from kidney tissue and HK-2 cells using the RNeasy separation kit (Qiagen, Valencia, CA, USA). After the concentration of RNA was determined, the total RNA was reverse transcribed into the cDNA using the Bio-Rad kit according to the manual. Primer (Table S1) amplification was carried out under the following conditions: denaturation at 95 °C for 20 s, annealing at 58 °C for 20 s, and extension at 72 °C for 20 s. Each primer was amplified for 40 cycles. The ratio for the mRNA of other genes was normalized to β-actin and presented as the mean ± SEM.

### 4.6. Western Blot Analysis

Protein was extracted from HK-2 cells and the kidney tissue of mice by radioimmunoprecipitation (RIPA)-Buffer (Beyotime, Jiangsu, China). A bicinchoninic acid (BCA) protein quantization kit (Beyotime, Jiangsu, China) was used to determine protein concentration. Proteins were separated by 10% sodium dodecyl sulfate-polyacrylamide gel electrophoresis (SDS-PAGE) and transferred to polyvinylidene fluoride membranes. The membranes were sealed with 5% milk for 2 h, incubated with KIM-1 (1:800), p-P65 (1:1000), RIPK1 (1:800), RIPK3 (1:800), cle-caspase-3 (1:1000), and β-actin antibodies (1:1000). This was incubated with IRDye 800-conjugated secondary antibody for 1.5 h at 25 °C. Finally, immunoreactive signals were detected with the LiCor/Odyssey infrared image system (LI-COR Biosciences, Lincoln, NE) and analyzed by Image J software (NIH, Bethesda, MD, USA). The experiment was repeated three times, using β-actin as an internal control.

### 4.7. Immunofluorescence

HK-2 cells were incubated overnight with eleutheroside B solution and cultured for 24 h in the presence or absence of cisplatin (20 μM). Cells were immobilized with 4% paraformaldehyde, incubated overnight with rabbit anti-KIM-1 (1:200) and anti-TNF-α (1:200) antibodies, and incubated with goat anti-rabbit IgG rhodamine for 1 h. Then, the cells were stained with 4′,6-diamidino-2-phenylindole (DAPI) and analyzed under a fluorescence microscope (Zeiss Spot; German Gottingen Karl Zeiss Microimaging Co., Ltd., Gottingen, Germany).

### 4.8. Periodic Acid-Schiff (PAS) Staining

The kidney tissues of mice were fixed with 10% formaldehyde for approximately 24 h, dehydrated, made transparent, and embedded in paraffin. The thickness of the sections was approximately 6 μm. After dewaxing, sections were stained with 1% periodic acid solution, and Schiff’s solution was added to the incubator at 37 °C. The nucleus was stained with hematoxylin for 5 min, and the sections were slowly washed with distilled water, dehydrated, and made transparent. After treatment, the sections were sealed with gum and photographed under a fluorescence inverted microscope.

### 4.9. Immunohistochemical Analysis

Kidney tissues were prepared into paraffin sections, dewaxed and pretreated, and incubated at room temperature for 10 min with goat serum blocking solution. Sections were incubated with rabbit anti-KIM-1 (1:500) and anti-TNF-α (1:500) antibodies overnight at 4 °C. Then, sections were incubated in biotin-labeled secondary antibody and liquid DAB (3,3′-diaminobenzidine tetrahydrochloride) Chromogen. After immunostaining, the slides were counterstained with hematoxylin. The prepared sections were photographed by the fluorescence inversion microscope and quantitatively analyzed by Image-Pro Plus software.

### 4.10. Flow Cytometry Analysis

The apoptotic rate was detected by flow cytometric analysis (BD FACSVerse, San Jose, CA, USA). HK-2 cells were treated with eleutheroside B for 12 h and incubated with 20 μM cisplatin for 24 h. HK-2 cells were washed twice with phosphate-buffered saline (PBS), digested with trypsin for 2 min, and centrifuged for 5 min at 1500 rpm. The apoptotic rate was detected according to the manufacturer’s instructions for the annexin V-fluorescein isothiocyanate/propidium iodide (AV-FITC/PI) apoptotic detection kit (Besby Company, Shanghai, China).

### 4.11. Gene Transcriptome Analysis Based on RNA-Seq Technology

HK-2 cells were incubated overnight with eleutheroside B solution and cultured for 24 h in the presence or absence of cisplatin (20 μM) [12,27]. Total RNA was extracted using a kit (Tiangen Company, Beijing, China). RNA-Seq technology was used to screen the differentially expressed genes after eleutheroside B treatment. Additionally, expression pattern and pathway analysis were performed for the differentially expressed genes, and candidates were selected for further expression variation validation.

### 4.12. Statistical Analysis

Image J software was used for quantitative analysis of protein bands, Excel software with Windows was used for real-time PCR analysis, and Image-Pro Plus software was used for quantitative analysis of fluorescent and immunohistochemical images. The data acquired from this study are presented as the mean ± SEM from 3–4 independent in vitro experiments on 6–8 mice. Statistical analyses were performed using the two-tailed unpaired t-test or one-way ANOVA, followed by the Newman–Keuls post hoc test (Prism 5.0; GraphPad Software, San Diego, CA, USA).

## Supplementary Materials

The following are available online. Supplementary Table S1: Primer pairs used for RT-PCR expression analysis.
