# Supplementary material for: Eleutheroside B Protects against Acute Kidney Injury by Activating IGF Pathway
Source: Molecules. 2019 Oct 28;24(21):3876. doi: 10.3390/molecules24213876 (PMC6864713; doi:10.3390/molecules24213876)
Supplement: Supplementary file 1 [file molecules-24-03876-s001.pdf]

The sequence of other primers used in the current study were listed as follows:

Table S1. The sequence of the primers.

	forward	reverse
mouse		
KIM-1	5'-CAGGGAAGCCGCAGAAAA-3'	5'-GAGACACGGAAGGCAACCAC-3'
TNF-α	5'-CATCTTCTCAAAATTCGAGTGACAA-3'	5'-TGGGAGTAGACAAGGTACAACCC-3'
MCP-1	5'-CTTCTGGGCCTGCTGTTCA-3'	5'-CCAGCCTACTCATTGGGATCA-3'
IL-6	5'-GAGGATACCACTCCCAACAGACC-3'	5'-AAGTGCATCATCGTTGTTCATACA-3'
β-actin	5'-CATTGCTGACAGGATGCAGAA-3'	5'-ATGGTGCTAGGAGCCAGAGC-3'
Human		
KIM-1	5'-CTGCAGGGAGCAATAAGGAG-3'	5'-TCCAAAGGCCATCTGAAGAC -3'
MCP-1	5'- CCAAAGAAGCTGTGATCTTCAA-3'	5'-TGGAATCCTGAACCCACTTC-3'
IL-6	5'- CGGGAACGAAAGAGAAGCTCTA-3'	5'- GAGCAGCCCCAGGGAGAA-3'
TNF-α	5'-CCCAGGGACCTCTCTCTAATCA-3'	5'-GCTACAGGCTTGTCACTCGG-3'
β-actin	5'-CGCCGCCAGCTCACCATG-3'	5'-CACGATGGAGGGGAAGACGG-3'
IGFBP-7	5'-AGCTGTGAGGTCATCGGAAT-3'	5'-GTCTGAATGGCCAGGTTGTC -3'
